# Second-line pharmacotherapy intensification after metformin monotherapy in type 2 diabetes: a nationwide register study from Finland during 2011–2022

**DOI:** 10.1186/s12913-024-11325-0

**Published:** 2024-08-19

**Authors:** Hanna Rättö, Terhi Kurko, Pia Pajunen, Helena Kastarinen

**Affiliations:** 1 Research Unit, The Social Insurance Institution of Finland, P.O. Box 450, Helsinki, 00056 KELA Finland; 2 Medical Advisory Unit, The Social Insurance Institution of Finland, Helsinki, Finland; 3 Medical Advisory Unit, The Social Insurance Institution of Finland, Kuopio, Finland

**Keywords:** Type 2 diabetes, Second line medicines, Pharmacotherapy, Intensification patterns, Reimbursement policy, Register study

## Abstract

**Background:**

In this nationwide register study, we examined the initiation of a second-line antidiabetic medicine (ADM) among new patients receiving regular metformin monotherapy in Finland during 2011–2022. We also reflected the second-line treatment patterns on changes in the reimbursement policy, and the national type 2 diabetes (T2D) care guidelines.

**Methods:**

Using register data on all reimbursed ADM purchases during 2010–2022, we defined nine annual cohorts of patients initiating regular metformin monotherapy during 2011–2019, each with a three-year follow-up. Descriptive methods were used to study the patterns of metformin monotherapy and second-line intensification over time. Proportional hazards models were used to analyse the take-up of the second-line ADM.

**Results:**

The share of new patients initiating metformin use (11–13% of all metformin users) and regular metformin use (83–85% of all new metformin users) remained stable. In all cohorts, 16–19% of the patients took up a second-line ADM (median time to intensification 1.5 years). With the 2011 cohort as reference, the highest proportion of new regular metformin users taking up a second ADM (hazard ratio 1.12. 95% confidence interval 1.07 ; 1.16, *P* < .0001) was in the 2019 cohort. In the 2017 cohort, the proportion of patients initiating sodium-glucose cotransporter 2 inhibitors as second-line treatment surpassed those initiating dipeptidyl peptidase-4 inhibitors. The reimbursement policy restricted the use of GLP-1-analogues.

**Conclusions:**

Second-line treatment intensification patterns over time paralleled the changes in the reimbursement system. Thus, our findings suggest that the reimbursement policy may influence the use of ADMs in Finland.

**Supplementary Information:**

The online version contains supplementary material available at 10.1186/s12913-024-11325-0.

## Background

The goals of type 2 diabetes (T2D) treatment are to prevent or delay complications and to maintain quality of life [1–3]. The repertoire of pharmacotherapies for T2D has increased in recent years [4]. A comparative study of 11 European countries found a rapid uptake of novel T2D medicines such as incretin-based therapy (glucagon-like peptide-1 (GLP-1) receptor agonists and dipeptidyl peptidase-4 (DPP-4) inhibitors) and sodium-glucose cotransporter (SGLT-2) inhibitors, suggesting an increase in the prescribing policy and prioritizing diabetes care as a healthcare priority [5].

The Finnish Current Care Guidelines for T2D [1] recommends metformin as the first-line anti-diabetic medicine (ADM) in most cases. In the choice of second-line treatment, the guidelines during the study period, for example the year 2016 edition of the guidelines, recommend accounting for the individual profile of the patient (Supplementary file 1). In addition to clinical considerations, the availability and costs of medicines can influence the choice of the second-line pharmacotherapy [6]. Results on T2D treatment intensification have previously been reported from some areas in Finland [7, 8], but national-level data are lacking.

In this nationwide register study, we examined the patterns of second-line ADM intensification among new patients initiating regular metformin monotherapy in Finland during 2011–2019. We used data on nine annual cohorts of new patients, each with a three-year follow-up, to compare the patterns of second-line pharmacotherapy intensification. We also examined if changes in the national reimbursement policy are reflected in the pharmacotherapy patterns.

## Methods

### Finnish context

All permanent residents in Finland are covered under the Finnish National Health Insurance (NHI) system, implemented by the Social Insurance Institution of Finland (Kela) [9]. Patients are entitled to reimbursements for outpatient medicines confirmed as reimbursable under the NHI scheme. In addition to the category of basic reimbursement (40% of the product price), there are two disease-based special reimbursement categories: (1) reimbursement at a higher special rate (100% with a copayment of €4.50/purchase) applied to e.g. medicines for cancer and epilepsy, and (2) reimbursement at a lower special rate (65% of the product price) applied to e.g. medicines for T2D and coronary artery disease. The reimbursability of a medicine can also be limited to apply to a specific diagnostic subgroup or for limited indications, or it can apply to certain treatment lines only, e.g. only after specified prior treatments have failed. Reimbursability of novel pharmaceuticals is often limited. Entitlement for special and limited reimbursements require a medical certificate. In addition to reimbursement categories, the annual maximum of copayments (€592 in 2022) aims to protect patients from very high copayments. (See [10] for more detailed information on the Finnish reimbursement system.)

During the study period, the national Current Care Guidelines for the treatment of T2D in adults [1] has recommended metformin monotherapy as the first-line pharmacological therapy in most clinical situations. However, over the past decade, there have been multiple changes in the recommendations related to second-line treatments, but not occurring during the study period between years 2017–2022 (Table 1). Current Care Guidelines for T2D was published for the first time in 2007, and have since been updated in 2009, 2013, and 2016, with the latest update in 2024. (Table 1).

**Table 1:** Timeline describing the development of the reimbursement policy patterns of T2D medicines in Finland and the publications and updates of the national Current Care Guidelines on the management of T2D in adults

Year	Development of reimbursement policy and the national care guidelines for T2D
2009	Publication of the first national *Finnish Current Care Guidelines* on the management of T2D in adults: second line recommendations, medicines equivalent to each other: glinides, DPP4is*, glitazones, SU*, insulins in severe hyperglycaemia. Of these medicines, all except DPP4is were reimbursed in special rate reimbursement (100%) category.
2010	DPP4is special rate *reimbursement* (100%) (unlimited for T2D).
2011	Update to the national *Finnish Current Care Guidelines* on the management of T2D in adults: second line recommendations, medicines equivalent to each other: glinides, DPP4is, glitazones, SU, insulins in severe hyperglycaemia.
2013	Update to the national *Finnish Current Care Guidelines* on the management of T2D in adults: the second line recommendations, medicines equivalent to each other: glinides, DPP4is, GLP-1RA*, pioglitazone, SU, insulins in severe hyperglycaemia. (See Note for GLP-1RA reimbursement)
2016	SGLT2is special rate *reimbursement* (100%) (unlimited for T2D), Update to the national *Finnish Current Care Guidelines* on the management of T2D in adults: second line recommendations, medicines equivalent to each other: glinides, DPP4is, GLP-1RA, pioglitazone, SGLT2i*, SU, insulins in severe hyperglycaemia.
2017	Lowering of the special *reimbursement* rate of all T2D medicines except insulins (from 100–65%).
2021	Limited special *reimbursement* rate (65%) for some GLP-1RA products (change in the reimbursement policy from third line to second line treatment, reimbursement still limited to treatment of obese individuals failing to achieve glycemic targets with first or second line treatment).

Note: The first GLP-1RA reimbursable at the limited basic rate (40%) (third line treatment) already in 2011. Several changes in reimbursement terms and in medicines related to GLP-1RA in third line treatment during 2011–2021

National Finnish Current Care Guidelines on the management of T2D in adults: recommendations for newly diagnosed T2D. *DPP-4i = dipeptidyl peptidase-4 inhibitors (gliptins); GLP-1RA = glucagon-like peptide 1 receptor agonists; SGLT2i = sodium-glucose cotransporter 2 inhibitors, SU = sulfonyl ureas

### Register data

We used administrative register data on all ADM purchases reimbursable under the NHI scheme and recorded in the Dispensations reimbursable under the NHI scheme register [11] maintained by Kela. From the register, we extracted information on all ADM purchases (the Anatomical Therapeutic Chemical (ATC) classification category A10) [12] made between January 2010 and December 2022. We collected information on the purchase date, the ATC-category of the medicine and a unique pseudonymised patient identifier as well as age and sex of the patients purchasing ADMs.

As the study was based solely on register data, no ethics approval or patient consent was needed according to Finnish legislation. All data were fully pseudonymised before we accessed them. As the register holder, Kela approved the use of the data for the current study.

### Annual cohorts and research design

Individuals purchasing reimbursed medicines belonging to ATC-category A10B were defined as T2D patients. Patients with no reimbursed purchases of any ADMs during the year prior to their first metformin (ATC A10B02) purchase were defined as new metformin users. Of them, patients who purchased metformin again within 180 days and did not purchase other ADMs within 180 days of their first metformin purchase were defined as new patients initiating regular metformin monotherapy. Based on the year of the first metformin purchase, we defined nine cohorts of new patients initiating regular metformin monotherapy, i.e. the annual cohorts for years 2011–2019. For the patients in these nine cohorts, the (possible) uptake of a second-line ADM during a three-year follow-up was studied. Patients purchasing a non-metformin ADM after 180 days of metformin monotherapy were defined as taking up a second-line ADM during follow-up. The first non-metformin ADM purchased after the 180 monotherapy was defined as the second-line ADM. (See also Supplementary file 2.)

We used five medicine groups when analysing the ADMs used in the second-line treatment intensification. SGLT2is, DPP-4is (i.e. gliptins) and GLP-1RAs were analysed separately. All insulins were analysed together as Insulins. Other ADMs (glinides, glitazones and sulfonylureas) were analysed together as Other medicines. The categorisation, including the handling of combination products and ATC -code alterations, is presented in more detail in Supplementary file 3.

### Statistical methods

Descriptive methods were used to study the treatment patterns over time. Cox proportional hazards model evaluating the share of followed-up units ‘at risk’ for the event of interest at any given time [13] was used to study the treatment intensification in the nine cohorts. In the proportional hazards analysis, patients initiating regular metformin monotherapy were followed for a maximum of 3 years to define uptake of a second-line ADM. Patients who did not initiate a second ADM during the maximum follow-up, were marked as censored. Patient’s sex and age at metformin initiation were used as controls in the analysis, and Wald’s test was used to test the statistical significance. Statistical analyses were performed using SAS version 9.4 [14].

## Results

Descriptive results for ADM treatment patterns in different annual cohorts are presented in Table 2. Of all metformin users, the proportion of new metformin users was 11–13% during the study period. Of all new metformin users, the proportion of patients remaining on regular metformin monotherapy varied between 83 and 85%. In all cohorts, approximately 16–19% of the patients took up a second-line ADM during the three-year follow-up. Median time to second-line treatment intensification varied from 1.49 years in the 2015 cohort to 1.64 years in the 2019 cohort.

**Table 2:** ADM treatment patterns in in nine annual cohorts of new metformin (MET) users

Annual cohort	All MET users *n*=	New MET users		New regular MET users		New regular MET users taking up 2nd ADM during 3-year follow-up		Median time to intensification in years (25 percentile; 75 percentile)
		*n*=	%*	*n*=	%**	*n*=	%***	
2011	225,103	28,404	*12.62*	23,595	***83.07***	3,891	*16.49*	1.50 (0.93;2.19)
2012	228,481	27,555	*12.06*	22,964	***83.34***	3,777	*16.45*	1.54 (0.96;2.23)
2013	233,492	28,262	*12.10*	23,458	***83.00***	3,700	*15.77*	1.53 (1.45;2.25)
2014	237,830	26,952	*11.33*	22,371	***83.00***	3,663	*16.37*	1.55 (0.97;2.28)
2015	242,440	26,188	*10.80*	21,648	***82.66***	3,612	*16.69*	1.49 (0.96;2.17)
2016	247,023	27,024	*10.94*	22,376	***82.80***	3,580	*16.00*	1.57 (0.98;2.29)
2017	252,991	28,491	*11.26*	24,107	***84.61***	3,938	*16.34*	1.60 (0.97;2.27)
2018	262,023	29,446	*11.24*	24,580	***83.47***	4,160	*16.92*	1.61 (0.99;2.29)
2019	269,983	29,708	*11.00*	24,834	***83.59***	4,571	*18.41*	1.64 (0.99;2.34)

MET = Metformin ; ADM = Anti-diabetes medicine. *of all MET users. ** of all new users. *** of all new regular users

Results for Cox proportional hazards modelling indicated some differences (Wald’s test *p* < .001) between cohorts in the take-up of the second-line ADM (Table 3). Compared to the cohort initiating regular metformin use in 2011, the hazard was significantly smaller (*p* < .05) with patients initiating regular metformin in 2013 (hazard ratio HR 0.949, 95% confidence interval CI 0.909; 0.995) and significantly higher (HR 1.115, 95% CI1.068; 1.163) in patients initiating regular metformin use in 2019. In the other annual cohorts, there were no significant differences compared to the 2011 cohort.

**Table 3:** Results for Cox proportional hazards modelling studying the differences between cohorts on the take-up of second ADM (with 2011 as reference cohort)

	Hazard ratio	95% Wald Cl	*P*
2011	1	reference group	
2012 (vs. 2011)	0.99	0.95; 1.04	NS
2013 (vs. 2011)	0.95	0.91 ; 0.99	*p* < .05
2014 (vs. 2011)	0.99	0.94 ; 1.03	NS
2015 (vs. 2011)	1.01	0.95 ; 1.03	NS
2016 (vs. 2011)	0.96	0.92 : 1.01	NS
2017 (vs. 2011)	0.98	0.94 ; 1.02	NS
2018 (vs. 2011)	1.02	0.98 ; 1.06	NS
2019 (vs. 2011)	1.12	1.07; 1.16	< 0.0001

Age (*p* < .001) and sex (*p* < .001) included as controls.

**Fig. 1:**
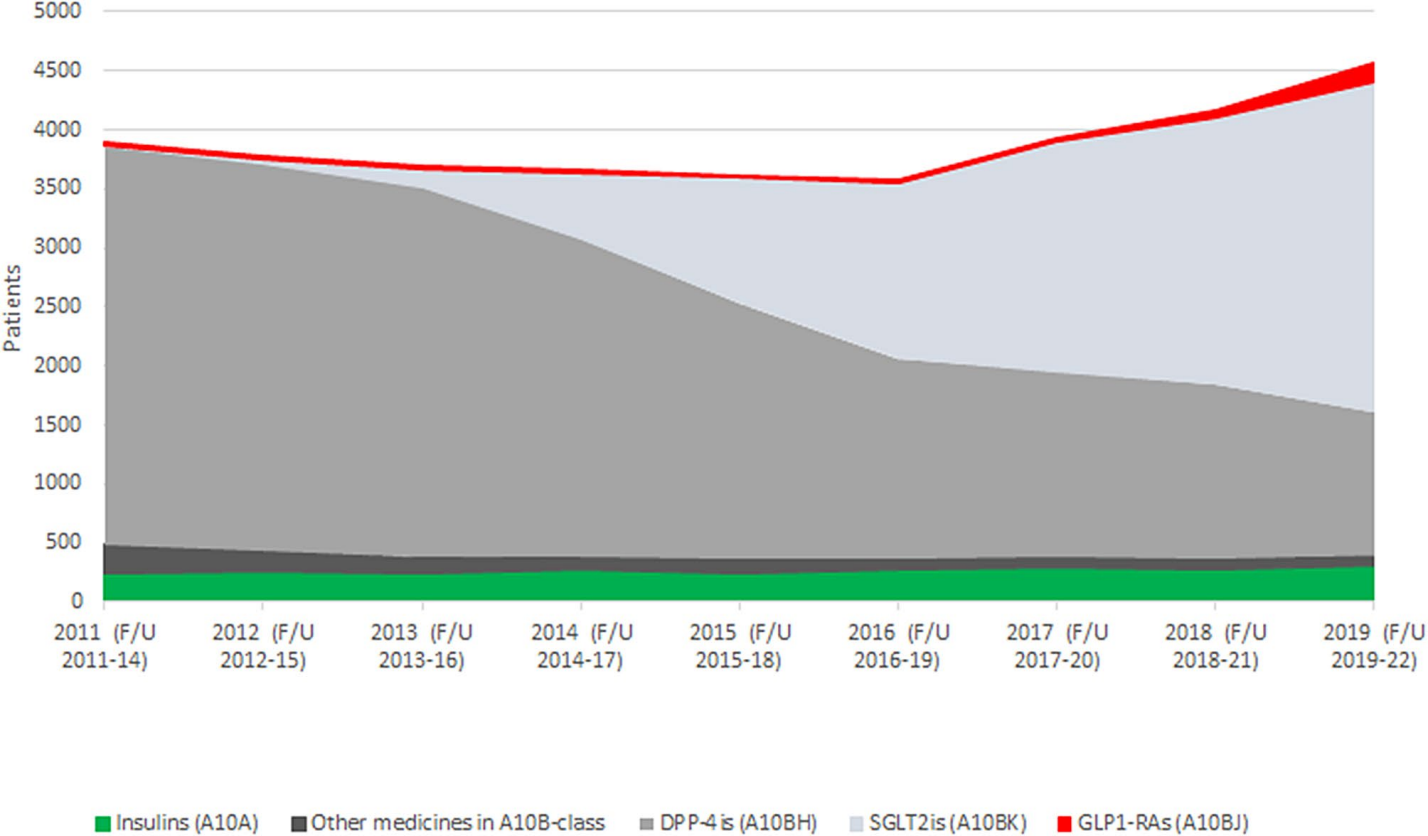
The uptake of a second ADM, by medicine group, for the new metformin users

Figure 1 presents the uptake of second ADM during the three-year follow-up by ADM-group for each cohort. The uptake of DPP-4is as the second ADM decreased and the uptake SGLT2is increased over the study period. The 2017 cohort was the first cohort, where the uptake of SGLT2is as the second ADM was larger than the uptake of DPP-4is. The share of GLP-1RAs as the second ADM remained small throughout the study period, though it was slightly higher in the 2019 cohort compared with the previous cohorts. (See also Supplementary file 4.) Only a small number of patients started older ADMs, i.e. glinides, glitazones or sulfonylureas (category Other medicines). The share of insulin users remained stable during the study period.

## Discussion

We examined the nationwide patterns in the second-line ADM initiation among patients initiating regular metformin monotherapy in Finland between 2011 and 2019. We used nine annual cohorts, each with a three-year follow-up period, to study the uptake of second-line ADMs. Our results show notable changes in the second-line treatment patterns over time, paralleling the changes in the reimbursement system and clinical guidelines during the study period.

Across the annual cohorts, the patterns of metformin use remained relatively stable. This held true for the proportion of new metformin users (out of all metformin users) as well as for the proportion of new regular metformin users. In all cohorts, approximately 16–19% of the patients took up a second ADM during the three-year follow-up. The median time to treatment intensification (1.5 years) is shorter than previously reported by Persson et al. [15] (around 5 years in 2015), but this can be explained by differences between the studies in defining the study population as well as in the criteria used for reporting the second-line intensification.

Proportional hazards modelling indicated that, when compared to the first cohort (2011), the hazard related to treatment intensification did not differ between the cohorts with the exception of cohorts 2013 and 2019. The follow-up of the 2013 cohort covers the period when the newer ADMs (DPP-4is, SGLT2is, GLP-1RAs) entered the market and were recommended as second-line treatment options in the national care guidelines. However, only DPP-4is were reimbursed in the special reimbursement category during that time. In the 2019 cohort, the proportion of new regular metformin users taking up a second ADM was the highest. Notably, by the end of the follow-up period, all recommended treatment options were reimbursed as second-line treatment. It should be also noted that the follow-up period of the 2019 cohort covered the COVID-19 era, which could be reflected in the second-line treatment intensification patterns, such as the median time to treatment intensification. A recent study investigating essential healthcare usage among T2D patients in the North Karelia region of Finland [16, 17] reported that after initial problems, diabetes care remained continuous despite the COVID-19 pandemic.

In the national Finnish Current Care Guidelines, the ADMs recommended in the second-line are considered equivalent to each other. The choice of the second-line medicine should be based on the needs and clinical profile of the patient. During the study period of 2011–2022, different ADMs have, however, entered the reimbursement system at different times, and the existing reimbursement criteria have changed in various ways and stages. Accordingly, the results of the current study showed the patterns of second-line treatments varying in time, especially with regard to the most used second-line ADM. DPP-4is, which were specially reimbursed during the whole study period, were the pivotal second-line T2D treatment in the beginning of the study period. This finding aligns with the findings by Persson et al. [15]. However, in the 2017 cohort, the proportion of patients initiating SGLT‐2is as the second-line treatment surpassed the proportion of patients initiating DPP‐4is, coinciding the change in the reimbursement status of the SGLT-2is in 2016. The use of GLP‐1RAs in second-line treatment intensification was minimal throughout the study period. However, the share of GLP‐1RAs in second line was slightly increasing in the last cohort compared with previous cohorts. This is likely explained by the fact that GLP-1RAs were only reimbursed in the second-line treatment after March 2021 (with limited reimbursement) before which they were reimbursed only as third-line treatment. Consistent with a previous report [18], the annual rates of intensification with older ADMs, such as sulfonylureas or glinides, remained small. The same was true for insulins.

These findings suggest that, in addition to clinical guidelines, the reimbursement policy may influence the choice of new T2D medicines used in the second-line treatment intensification in Finland. This is in line with data from the United Kingdom [19] reporting that the treatment options used at the first stage of treatment intensification for T2D are not driven by clinical need alone. Results also parallel previous findings from Finland that have implied changes in reimbursement policy being reflected in the use of new antiglaucoma medicines and statins [20, 21].

It should also be noted that in 2017, all antidiabetic medicines, except insulins, were transferred from the higher special reimbursement category (100%) to the lower special reimbursement category (65%). However, despite this major change in the national reimbursement policy, aimed to decrease the reimbursement expenditure of ADMs, the progressive increase of initiation of the SGLT-2is in the second line following their entrance to the special rate reimbursement category has continued. In agreement with previous findings [22], our results show, that the treatment of T2D in Finland has been continuously changing towards newer treatments.

The majority of T2D patients in Finnish primary care are at a very high risk for T2D complications, especially cardiovascular events [23]. Scientific evidence has shown that SGLT2is or GLP-1RAs can improve cardiovascular outcomes as well as secondary outcomes, such as heart failure and progression of renal disease, in patients with established cardiovascular or chronic kidney disease [2, 24, 25], the most costly complications of T2D [26, 27]. New evidence implies that T2D care should be targeted even more according to the individual characteristics of the T2D patients [28].

In Finland, some but not all of the novel medicines have been included in the reimbursement scheme relatively soon after their marketing authorisation (Table 1). The awareness of T2D in Finland has also been increased by nationwide programs during the early 2000’s [29]. However, alarmingly, recent data from the North Karelia region of Finland observed that achieving glucose target has deteriorated even after T2D treatment has been intensified [30]. Further studies are needed to evaluate the benefits of newer ADMs in clinical practice. These data may be available in the future, as the new Finnish National Diabetes Register officially started in the beginning of year 2023 [31].

Strengths of the present study include the population-based, nationwide, and unselected real‐world design with recent data covering all reimbursed ADMs purchased between 2011 and 2022. The reliability and suitability of Finnish administrative registers for epidemiological studies have repeatedly been verified [32, 33]. Additionally, to include patients who have not applied for entitlement to special reimbursements, we used information on all reimbursed ADMs to define the study population. As metformin treatment is relatively cheap, patients may not always apply for the entitlement if metformin is their only T2D medicine.

The limitations of the study should be noted. Dispensations reimbursable under the NHI scheme register does not contain data on ICD-codes or any anthropometric, clinical, or laboratory variables. Thus, we cannot rule out the possibility that prediabetic individuals, patients with some other type of diabetes, or patients using metformin for other health condition, might be included in this analysis. Nevertheless, it is probable that initiating a second-line T2D treatment would have indicated or verified a T2D diagnosis in most of these cases. The proportion of patients with an unrelated health condition (such as polycystic ovaries) is likely to be minimal. In addition, Finnish Current Care guideline [1] recommends that the patient’s individual profile should be taken into account when selecting treatments. However, due to limited information recorded in the utilised register we were unable to assess the data related to individual clinical characteristics such as HbA1c-level or BMI that would affect the treatment patterns. As the data only cover reimbursable medicine purchases, we miss, for example, the possible use of GLP-1RAs without any reimbursement in T2D treatment. However, due to their high costs, this is not likely to be in large scale. As the reimbursement of liraglutide used for obesity indication is restricted to non-diabetic individuals and semaglutide was not in market for obesity indication in Finland during the study period, these are not considered marked confounding factors in the present study. Finally, the data do not contain information on treatment adherence after the purchase.

The aim of this study was to provide a general level nationwide understanding of the medicine use intensification patterns in T2D. However, in future studies it would be valuable to assess individual-level factors associated with medication intensification patterns. The Finnish National Diabetes Register, containing individual level clinical and administrational data, enables more detailed studies on diabetes medicine usage in the future. In addition, due to the rapid development of GLP-1-RA’s it would be valuable to conduct a nationwide study to assess their patient-level use and intensification patterns in different indications.

In conclusion, the patterns of metformin use and the share of patients taking up a second-line treatment remained relatively stable during 2011–2022. However, notable changes were observed in the pivotal second-line treatments. Changes in the reimbursement policy were reflected in the second-line treatments, most notably in the uptake of SGLT-2is. The use of GLP‐1RAs as the second-line treatment was minimal, reflecting the limited reimbursement status. Even though our research design does not allow drawing conclusions on causality, our data indicate that the attributes of the reimbursement system may influence the selection of the second-line ADMs in Finland.

### Electronic supplementary material

Below is the link to the electronic supplementary material.

Supplementary Material 1

Supplementary Material 2

Supplementary Material 3

Supplementary Material 4

## Data Availability

Due to data protection regulations of the secondary use of administrative, individual-level register data in Finland, the authors do not have the permission to make the data supporting the current findings available [34]. Interested parties may however apply for permission to access the data from the Social Insurance Institution of Finland (Kela), https://tietotarjotin.fi/en/instructions/2714327/how-to-apply-for-data-permit-from-kela; email: tietoaineistot@kela.fi; tel.:+358504762974.
