# Supplementary material for: Second-line pharmacotherapy intensification after metformin monotherapy in type 2 diabetes: a nationwide register study from Finland during 2011–2022
Source: BMC Health Serv Res. 2024 Aug 19;24:944. doi: 10.1186/s12913-024-11325-0 (PMC11331595; doi:10.1186/s12913-024-11325-0)
Supplement: Supplementary file 1 — Supplementary Material 1 [file 12913_2024_11325_MOESM1_ESM.pdf]

**Supplementary table 1**. Characteristics of individual patient profile that should be taken into account when selecting second line treatment option according to the Finnish Current Care Guideline of Diabetes* [1]

| **Individual characteristics mentioned in the Current Care Guideline of Diabetes (2016)** |
|---|
| Early type 2 diabetes |
| Chronic type 2 diabetes (more than 10 years) |
| Type 2 patient with obesity |
| Older adult with type 2 diabetes |
| Commercial vehicle driver with type 2 diabetes |
| Type 2 diabetes patient with chronic kidney disease |
| Level of hyperglycemia in patient with type 2 diabetes |

*To note: There were no major updates in the Finnish Current Care Guidelines for type 2 diabetes, during the study period, between years 2017 and 2022.
