# Supplementary material for: Second-line pharmacotherapy intensification after metformin monotherapy in type 2 diabetes: a nationwide register study from Finland during 2011–2022
Source: BMC Health Serv Res. 2024 Aug 19;24:944. doi: 10.1186/s12913-024-11325-0 (PMC11331595; doi:10.1186/s12913-024-11325-0)

Supplementary file 2. Flow chart for regular metformin monotherapy initiators, year 2011 as an example. All data linkage is done with pseudo-identifiers. ATC= Anatomical Therapeutic Chemical

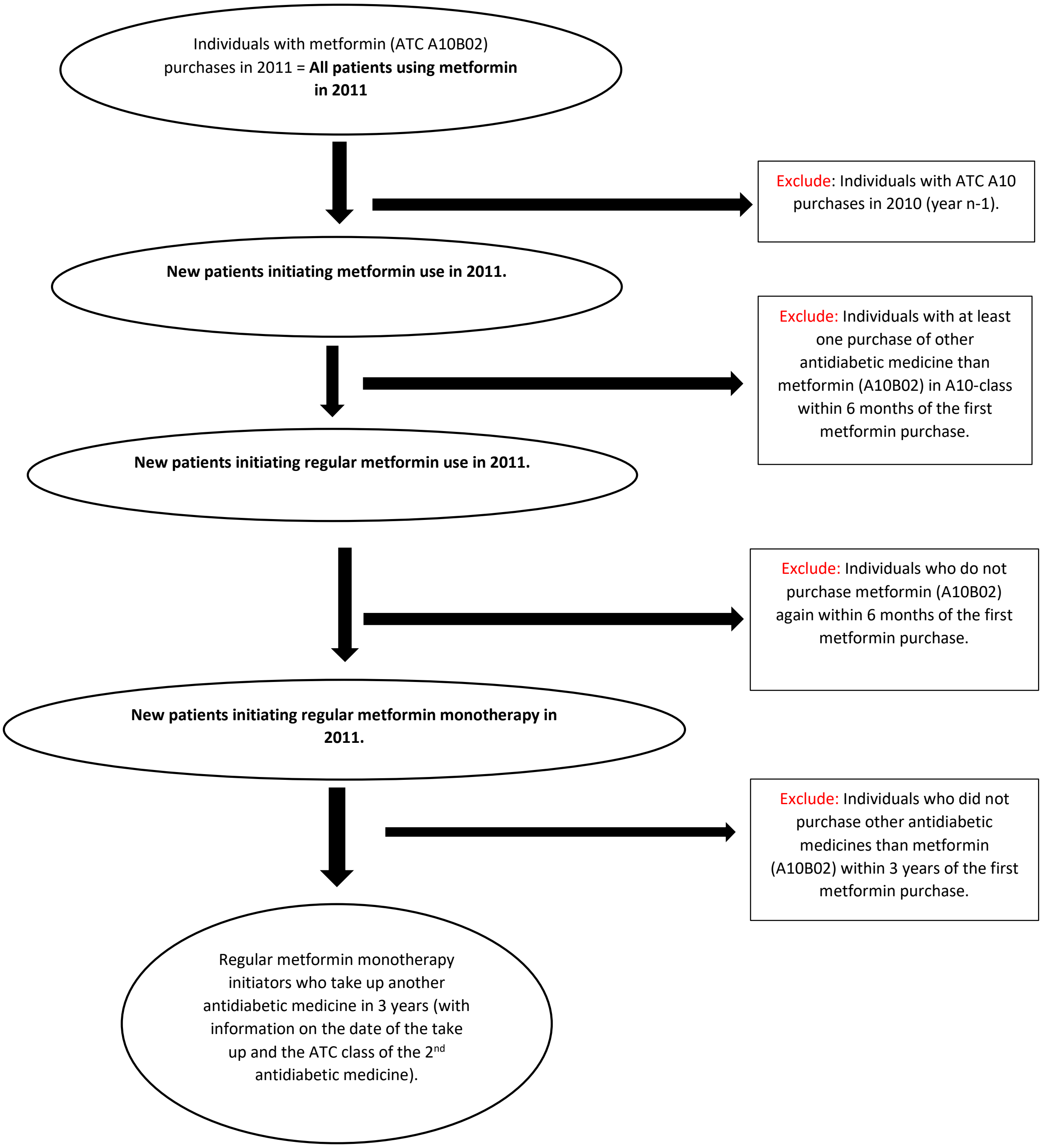

Supplement: Supplementary file 2 — Supplementary Material 2 [file 12913_2024_11325_MOESM2_ESM.pdf]
