# Supplementary material for: Second-line pharmacotherapy intensification after metformin monotherapy in type 2 diabetes: a nationwide register study from Finland during 2011–2022
Source: BMC Health Serv Res. 2024 Aug 19;24:944. doi: 10.1186/s12913-024-11325-0 (PMC11331595; doi:10.1186/s12913-024-11325-0)
Supplement: Supplementary file 3 — Supplementary Material 3 [file 12913_2024_11325_MOESM3_ESM.pdf]

Supplementary file 3. Analysed medicine classes and their classification

	Medicine group (ATC-group, 4th level, chemical subgroup)	Individual medicines in use in Finland (ATC-code)	How are recoded in the analysis
Insulins and analogues (A10A)			
	Insulins and analogues for injection, fast-acting (A10AB)<br>Insulins and analogues for injection, intermediate-acting (A10AC)<br>Insulins and analogues for injection, intermediate- or long-acting combined with fast-acting (A10AD)<br>Insulins and analogues for injection, long-acting (A10AE)		**Insulins**
Blood glucose lowering drugs, excluding insulins (A10B)			
	Biguanides (A10BA)	metformin (A10BA02)	**Metformin**
	Sulfonylureas (A10BB)	glimepiride (A10BB12)	**Other medicines**
	Combinations of oral blood glucose lowering drugs (A10BD)	metformin and sitagliptin (A10BD07)<br>metformin and vildagliptin (A10BD08)<br>pioglitazone and alogliptin (A10BD09)<br>metformin and saxagliptin (A10BD10)<br>metformin and linagliptin (A10BD11)<br><br>metformin and dapagliflozin (A10BD15)<br>linagliptin and empagliflozin (A10BD19)<br>metformin and empagliflozin (A10BD20)<br>saxagliptin and dapagliflozin (A10BD21)<br>metformin and ertugliflozin (A10BD23)<br>sitagliptin and ertugliflozin (A10BD24)	**DPP-4is**<br>**DPP-4is**<br>**DPP-4is**<br>**DPP-4is**<br>**DPP-4is**<br><br>**SGLT2**<br>**SGLT2**<br>**SGLT2**<br>**SGLT2**<br>**SGLT2**<br>**SGLT2**
	Thiazolidinediones (A10BG)	pioglitazone (A10BG03)	**Other medicines**
	Dipeptidyl peptidase 4 (DPP-4is) inhibitors (A10BH)	sitagliptin (A10BH01)<br>vildagliptin (A10BH02)<br>saxagliptin (A10BH03)<br>alogliptin (A10BH04)<br>linagliptin (A10BH05)	**SGLT2**
	Glucagon-like peptide-1 (GLP-1) analogues (A10BJ)	exenatide (A10BJ01)<br>liraglutide (A10BJ02)<br>lixisenatide (A10BJ03)<br>dulaglutide (A10BJ05)<br>semaglutide (A10BJ06)	**GLP-1RA**
	Sodium-glucose co-transporter 2 (SGLT2) inhibitors (A10BK)	dapagliflozin (A10BK01)<br>canagliflozin (A10BK02)<br>empagliflozin (A10BK03)<br>ertugliflozin (A10BK04)	**SGLT2**
	Other blood glucose lowering drugs, excl. insulins (A10BX)	repaglinide (A10BX02)	**Other medicines**

* ATC= Anatomic-Therapeutic-Chemical-Classification
