# Supplementary material for: Second-line pharmacotherapy intensification after metformin monotherapy in type 2 diabetes: a nationwide register study from Finland during 2011–2022
Source: BMC Health Serv Res. 2024 Aug 19;24:944. doi: 10.1186/s12913-024-11325-0 (PMC11331595; doi:10.1186/s12913-024-11325-0)
Supplement: Supplementary file 4 — Supplementary Material 4 [file 12913_2024_11325_MOESM4_ESM.pdf]

Supplementary file 4.

The proportion of patients initiating SGLT2is as second-line treatment, compared with the proportion of patients initiating DPP4s as second-line treatment, by annual cohort. Means and 95% confidence intervals (CI).

	Mean, 95% CI
2011	0.00 (0.000.00)
2012	0.01 (0.01; 0.01)
2013	0.05 (0.04; 0.05)
2014	0.17 (0.16; 0.18)
2015	0.33 (0.31; 0.34)
2016	0.47 (0.45; 0.48)
2017	0.55 (0.54; 0.57)
2018	0.61 (0.59; 0.62)
2019	0.70 (0.68; 0.71)

The proportion of patients initiating GLP-1RAs as the second-line treatment by annual cohort. Means and 95% confidence intervals (CI).

	Mean, 95% CI
2011	0.01 (0.00; 0.10)
2012	0.01 (0.00; 0.01)
2013	0.01 (0.01; 0.01)
2014	0.01 (0.01; 0.01)
2015	0.01 (0.00; 0.01)
2016	0.01 (0.01; 0.01)
2017	0.01 (0.01; 0.01)
2018	0.01 (0.01; 0.02)
2019	0.04 (0.03; 0.04)
